# Gender differential on characteristics and outcome of leprosy patients admitted to a long-term care rural hospital in South-Eastern Ethiopia

**DOI:** 10.1186/1475-9276-11-56

**Published:** 2012-10-04

**Authors:** José M Ramos, Miguel Martínez-Martín, Francisco Reyes, Deriba Lemma, Isabel Belinchón, Félix Gutiérrez

**Affiliations:** 1 Gambo General Rural Hospital, Shashemane, Ethiopia; 2 Service of Internal Medicine, Hospital General Universitario de Alicante, Pintor Baeza, Alicante, 12 03010, Spain; 3 Department of Clinical Medicine, Universidad Miguel Hernández, Alicante, Spain; 4 Service of Internal Medicine, Hospital La Princesa, Madrid, Spain; 5 Service of Dermatology, Hospital General Universitario de Alicante, Alicante, Spain; 6 Infectious Diseases Unit, Hospital General Universitario de Elche, Alicante, Spain

**Keywords:** Leprosy, Gender, Sex, Female, Hospital, Ethiopia

## Abstract

**Introduction:**

In previous studies, women are less aware of causation and symptoms of leprosy and have less access to health care coverage than men, thus contributing to their delay in seeking for treatment. We assess the gender differences in leprosy cases admitted to a rural referral hospital in Ethiopia for 7 and a half years.

**Methods:**

Retrospective data of the leprosy patients admitted to referral hospital were collected using leprosy admission registry books from September 2002 to January 2010. Variables were entered in an Excel 97 database.

**Results:**

During the period of study, 839 patients with leprosy were admitted; 541 (64.5%) were male, and 298 (35.6%) female. Fifteen per cent of female patients, and 7.3% of male patients were paucibacillary leprosy cases while 84.8% of female patients and 92.7% of males were multibacillary leprosy cases (p<0.001). Female leprosy patients were younger than male ones (median: 36 versus 44 years) (p<0.001). In the multivariate analysis, age (odds ratio [OR]: 0.97; 95% confidence interval [CI]: 0.96-0.98; p<0.001), admission for cardiovascular diseases (OR: 7.6, 95% CI: 1.9-29.3; p=0.004), admission for gastroenteritis (OR: 14.0; 95% CI: 1.7-117; p=0.02), admission from out patients clinic (OR: 2.04; 95% CI: 1.1-4.01; p=0.02), and mortality as final outcome (OR: 3.1, 95% CI: 1.2-8.0; p=0.02) were independently associated with female gender.

**Conclusions:**

Female patients with leprosy admitted to hospital were younger, had a different profile of admission and a higher mortality rate than male ones.

## Introduction

Leprosy is a chronic disease with low-grade infectivity caused *by Mycobacterium leprae,* that can lead to deformities, physical handicap, and social stigma [1]. During the 1990s and 2000s, leprosy services were intensified in a World Health Organisation (WHO) stimulated effort for reducing the burden of disease in many endemic countries. A consequence of this is that the number of registered leprosy cases has decreased from 5 351 408 cases in 1985 to 926 259 cases in 1996 and 244 796 cases in 2009 [2-4]. The introduction of multi-drug therapy (MDT) made possible that leprosy patients could be diagnosed, treated and followed-up in outpatient clinics. However, referral centres continue to play a key role in providing specialized services for patients with complications of the disease, such as leprosy reaction, neuropathy or skin ulcer, as well as in the prevention of disabilities and rehabilitation. Referral centres that are part of the general health-care system have also been crucial in helping primary care [4].

In two cohorts of newly detected leprosy patients in Bangladesh (The Bangladesh Acute Nerve Damage Study [BANDS]) and Ethiopia (ALERT MDT Field Evaluation Study [AMFES]), there were fewer women diagnosed of leprosy than men (ratio female/male 0.6) [5]. Moreover, women are less aware of causation and symptoms of leprosy and have less access to health care coverage than men, thus contributing to their delay in seeking for treatment [6-8]. Thus, understanding gender differences in clinical presentation, type of leprosy, and access to health of leprosy patients is important [9]. Also, female leprosy patients suffer more isolation and rejection from society than males [1,10-12].

In this study, we analyse gender differences in clinical and epidemiological characteristics and outcomes of leprosy patients admitted to a long-term care rural referral hospital in South-eastern Ethiopia.

## Methods

### Setting

The Gambo Rural Hospital (GRH) is a rural hospital in Ethiopia (in the Oromiya region), which is a referral centre in the programme of leprosy care in the country according to the guidelines of Tuberculosis and Leprosy Prevention and Control Programme (TLPCP) [13,14]. The GRH is located in West-Arsi zone, 250 Km southeast of Addis Ababa. Most of the population live in a rural setting and work in agriculture and farming. In 1961 the Leprosy Centre in Gambo begins its work. Later on (1966–1970), the National Policy for Leprosy Services changes from Leprosy Settlement to Leprosy Control Units or Stations. Gambo Leprosy Centre becomes an Integrated Health Unit catering to all health needs, including leprosy patients, with a 49 beds dispensary. In 1985, the Gambo Leprosy Control Centre changes its name to GRH. Several leprosy cases treated in GRH continued living around the hospital in Gambo leprosy village (about 250 families).

The diagnosis of leprosy is usually made with clinical findings according to TLPCP [9,10]. According of TLPCP, criteria for admission are severe erythema nudoso leprosum (ENL) reaction, deep skin ulcer, red and/or painful eye, pregnancy, tuberculosis or any other severe infection, age younger than 12, recent history of peptic ulcer in the stomach or duodenum, history of diabetes, general illness with fever, patients who did not improve during a previous course of treatment and patients who improved during previous course, but who develop a reaction for the third time [13,14].

In Ethiopia there is a main referral hospital, ALERT Hospital, in Addis Abeba where patients were transferred from different first level leprosy care and from peripheral referral leprosy hospitals [15]. There are 5 referral leprosy hospitals in Ethiopia and GRH is one of them.

All leprosy patients with medical and surgical problems who need hospital admission related or not with leprosy are admitted to the leprosy ward in GRH. The patients stay in hospital until recovery of their problems. The cost of hospitalization is free for them. The food is provided by the hospital. After being discharged, the patients may continue attending the GRH clinic or are transferred to other clinics near their homes.

### Study design and data collection

Diagnosis of leprosy is usually made in the GRH with clinical examination according to TLPCP [13,14]. Patients who are diagnosed as new or old leprosy cases at GRH are: 1) admitted to hospital in the case of seriously ill leprosy patients, 2) registered at the GRH leprosy clinic or 3) referred to leprosy clinics near their home. A retrospective data collection from all the patients diagnosed of leprosy and admitted to the leprosy ward was done using the admission registry book. Patients were classified according to the number of leprosy skin lesions and the result of the skin smear examination as: multibacillary (MB) leprosy (six or more skin lesions or less than six skin lesions which have a positive slit skin smear result) or paucibacillary (PB) leprosy (one to five leprosy skin lesions [13,14].

The health workers completed the admitting forms, which included variables of age, sex, place of residence, case definitions, WHO classification of leprosy (MB or PB leprosy), origin of admission, length of hospital stay, diagnosis during admission, and outcome.

Case definitions were: (1) new case: a patient with MB or PB leprosy who has never received treatment for leprosy before, (2) a patients who relapses after MDT: a patient properly treated with a complete course of MDT, but who returns to the health service and is found to have active leprosy again; (3) return after withdrawal: an MB patient who returns for treatment, after having missed more than 3 four-weekly doses of MDT; and (4) transfer in: a patient who started treatment in one health setting and moved to another to continue treatment. Origin of admission: field (if the patients was admitted from other leprosy clinic); outpatients (OPD) department (if the patients was admitted from the OPD department). Diagnosis during admission was made using standardised definitions. Definitions of outcome during admission: (1) improved: a patient who has improved within the admission period, (2) died: a patients who dies of any cause, (3) not improvement: a patient who does not improve, or (4) run away: a patient who leaves voluntarily the hospital.

The period analysed in the study began in September 2002 and ended in January 2010, (seven and a half years). Re-admission data were only available for admissions from 1^st^ December 2004 to 31^st^ January 2010. The ethics committee approvals were obtained from the local Research and Publication Committee of the GRH, and Health Unit and Ethical Review Committee of the Ethiopian Catholic Secretary.

### Statistical analysis

Epidemiological and clinical data were entered in an Excel 97 database. Statistical analysis were performed with the use of SPSS software, version 12. Continuous variables are given as median, range, and interquartile range (IQR). Chi-square test was used for comparison between two groups of subjects (male and female), Fisher’s exact test was used in the case the sample size of patients wasn`t large enough, and Kruskal-Wallis test, a nonparametric test, was used to compare the significance of the difference between the distributions of two independent samples. Each category was analysed in a univariate way. Univariate predictors with p-value < 0.25 were included in a logistic regression model to identify independent variables associated with gender by odds ratio (OR) with a 95% confidence interval (CI).

## Results

In the seven and a half years period, 839 patients with leprosy were admitted to GRH with an average of 100 cases per year. Among them, 541 (64.5%) were male, and 298 (35.6%) female. The male/female ratio of admitted patients was 1.8. Two hundred and forty four (29.1%) patients were living in the catchment area of the GRH. Female leprosy patients were living in the catchment area more than male leprosy patients (37.6% versus. 24.4%, p<0.001). The median age of study population was 40 years (IQR: 25 – 55). Twenty-seven (3.2%) were children (under 15 years old). Female leprosy patients were younger (median age: 36 years) than male ones (median age: 44 years; p<0.001) (Table 1). Forty-seven per cent in the female group, and 35.9% in the male group were included in the 19 to 35 age interval (p=0.005), and 39.9% of female patients, and 55.5% of male patients were older than 35 year-old (p<0.001).

**Table 1:** Median, range and interquartile range (IQR) of age and days of admission in female and male leprosy patients

	**Missing**	**Total**		**Male**		**Female**		**P value**
		**Median**	**Range/IQR**	**Median**	**Range/IQR**	**Median**	**Range/IQR**	
Age, years	M:0; F:0	40	4-81/25-55	44	9-81/28-57	36	4-77/24-50	<0.001
Duration of hospital stay, days	M: 16; F:13	65	2- 550/36-111	68	2-550/40-108	60	1- 490/28-113	0.69

M: male; F: female.

Data from re-admission were only available for 519 cases. There were 84 (16.2%) cases of readmission without differences by gender. The median of readmissions per patient was 2 times (range: 2–8). The main characteristics of these individuals by gender are shown in Table 2. Thirty nine percent of female lived and 24.4% of male lived in the catchment area (p<0.001). Fifteen per cent of female and 7.3% of male were PB leprosy cases while 84.8% of female, and 92.7% of male were MB leprosy cases (p<0.001). The prevalence of new MB, and new PB cases was similar in women and men (16.6% versus 13.2%, and 2.0% versus 1.1%, respectively). However, the prevalence of old MB cases was significantly lower in female (68.2%) than in male ones (79.6%), with old PB cases being more common in female ones (13.2%) than in male (6.1%) (p=0.001). There was no difference by gender in the prevalence of patients on MDT. Female leprosy patients were admitted from the OPD in 95.6% of the cases, and from the “field” in only 4.4%; among male leprosy patients, admission was made from the OPD in 93.8%, and from the “field” in 8.7% (p=0.03).

**Table 2:** Main epidemiological and clinical characteristics, and outcome in female and male leprosy patients

**Variables**	**Missing**	**Total**		**Male**		**Female**		**Chi- square value**	**P value**
		**N**	**%**	**N**	**%**	**N**	**%**		
Origin	M:0; F:0							16.2	<0.001
Catchment area		244	29.1	132	24.4	112	37.8		
Outside catchment area		595	70.9	409	75.6	186	62.2		
Age	M:0; F:0								
< 18		89	10.6	47	8.7	42	14.1	5.88	0.020
19-35		331	39.5	194	35.9	137	46.0	8.21	0.005
> 35		419	49.9	300	55.5	119	39.9	18.12	<0.001
Re-admission	M: 204; F: 114							0.09	0.82
Yes		84	16.2	53	15.8	31	16.8		
No		435	83.8	282	84.2	153	83.2		
WHO classification	M:1; F:2							13.49	<0.001
PB		84	10.0	39	7.3	45	15.2		
MB		752	90.0	501	92.7	251	84.8		
On MDT	M:1; F:2							2.68	010
Yes		132	15.7	78	14.3	55	18.6		
No		710	84.3	461	85.7	237	81.4		
Old or New cases	M:1; F:2								
New MB		120	14.3	71	13.2	49	16.6	1.81	0.21
New PB		12	1.4	6	1.1	6	2.0	0.02	0.99
Old MB		632	75.6	430	79.6	202	68.2	13.41	<0.001
Old PB		72	8.6	33	6.1	39	13.2	12.13	0.001
Admission from	M:0; F:0							5.39	0.03
OPD		779	92.8	494	91.3	285	95.6		
Field		60	7.2	47	8.7	13	4.4		
Outcome	M:37; F:21								
Improved		707	90.6	459	91.0	248	89.6	0.49	0.56
Referred		36	4.6	25	5.0	11	4.0	0.30	0.65
Death		22	2.8	10	2.0	12	4.3	3.60	0.09
Not improved		8	1.0	4	0.8	4	1.4	0.29	0.62
Voluntary discharged		8	1.0	6	1.2	2	0.7	0.05	0.80

M: male; F: female; MDT multidrug therapy; MB: multibacillary; PB: paucibacillary; OPD: outpatients department.

The main diagnosis after discharge was neuropathic skin ulcer (56.2%). The diagnosis of neuropathic skin ulcer was performed in 48.3% of women and 60.5% of men (p=0.001). The other mainly diagnoses during the admission were: reversal reaction, neuritis, osteomyelitis, ENL, and lower respiratory tract infection without differences by gender as shown in Table 3. Differences of prevalence between female and male were found in diagnosis of cases not related with leprosy, such as cardiovascular disease (3.4% versus 0.6%; p=0.004) or gastroenteritis (2.8% versus 0.1%; p=0.002), being the prevalence of these diseases very low in the sample.

**Table 3:** Diagnosis during hospital admission in female and male leprosy patients

			**Total**		**Male**			**Chi- square value**	**P value**
	**M:2; F:6**	**N**	**%**	**N**	**%**	**N**	**%**		
Infected skin ulcer		467	56.2	326	60.5	141	48.3	11.52	0.001
Reversal reaction		80	9.6	48	8.9	32	10.9	0.80	0.40
Neuritis		72	8.7	49	9.1	23	7.9	0.30	0.64
Osteomyelitis		68	8.2	47	8.7	21	7.2	0.10	0.81
ENL		46	5.5	29	5.4	17	5.8	0.03	0.91
LRTI		31	5.7	17	3.1	14	4.8	0.50	0.32
Cellulitis		16	1.9	9	1.7	7	2.4	0.30	0.64
Diabetes mellitus		13	1.6	9	1.7	4	1.4	0.04	0.97
Cardiovascular diseases		13	1.6	3	0.6	10	3.4	9.92	0.004
Gastroenteritis		9	1.1	1	0.2	8	2.7	11.29	0.002
Accident		7	0.8	4	0.7	3	1.0	0.20	0.70
Chronic liver diseases		7	0.8	2	0.4	5	1.7	3.1	0.06
Tuberculosis		6	0.7	5	0.9	1	0.3	0.30	0.60
Arrhythmia		5	0.6	4	0.7	1	0.3	0.35	0.66
Eyes problems		5	0.6	1	0.2	4	1.4	3.3	0.05
Peptic ulcer		5	0.6	1	0.2	4	1.4	3.2	0.06
Intestinal parasites		5	0.6	3	0.6	2	0.7	0.01	0.99
Mental disorders		4	0.5	1	0.2	3	1.0	2.5	0.12
Neoplasm		3	0.4	3	0.6	0	0.0	0.5	0.31
Urinary tract infection		3	0.4	3	0.6	0	0.0	0.5	0.30
Others		12	1.4	7	1.3	5	1.7	-	-
Total*		831	100	539	100	292	100	-	-

M: male; F: female; ENL: erythema nodosum leprosum; LRTI: lower respiratory tract infection.

* Because each patient could have >1 diagnosis, the number of cases can be higher than the number of patients.

The median length of hospital stay was 65 days (IQR: 36–111) (Table 1). There was no difference between both groups. The majority of patients recovered uneventfully (90.6%), with no statistical differences between men and women. Only 4.6% of the sample was referred to other hospitals. The hospital mortality was low (2.8%), slightly higher in female than in male patients (4.3% versus 2.0%; p=0.07). Other outcomes are shown in Table 3.

In the multivariate analysis, age (OR: 0.97; 95% CI: 0.96-0.98; p<0.001) was inversely associated with female gender, in contrast admission from the OPD (OR: 2.4, 95%: CI 1.1-4.0; p=0.03; versus field admission), and mortality as final outcome (OR: 3.1, 95% CI: 1.2-8.0; p=0.02) were directly associated with female gender (Table 4).

**Table 4:** Variables associated with female gender in a multivariate analysis*

	**OR**	**95% CI**	**P value**
Age	0.97	0.96 - 0.98	<0.001
Admission for cardiovascular diseases	7.6	1.9 - 29.3	0.004
Admission from the OPD	2.4	1.1 - 4.0	0.03
Admission for gastroenteritis	14.0	1.7-117	0.02
Died as final outcome	3.1	1.2 - 8.0	0.02

NOTE: OR: odds ratio; 95% confidence intervals (CI); OPD: outpatient clinic.

* Variables with p-value < 0.25 in univariate analysis were included in the logistic regression model. They were: origin of patients, age of patients WHO classification, old or new paucibacillary or multibacillary leprosy cases, admission from field or OPD, died as final outcome, and infected skin ulcer, cardiovascular diseases, gastroenteritis, chronic liver diseases, eyes problems, peptic ulcer, and mental disorder as cause of admission.

## Discussion

In Ethiopia, since the introduction of MDT for leprosy patients, leprosy control was incorporated as an aim within the general health service system [7]. A referral system could play a crucial role in ensuring the quality of services in an integrated leprosy control programme [13,14]. This study analyzed inpatients with leprosy in a rural referral hospital and found significant differences by gender.

Traditionally, the preponderance of male patients over female ones has been reported [5-8]. In the current study, the ratio male/female in admitted leprosy patients (1.8) was slightly lower than the observed in a new cases sample diagnosed in our centre previously (2.1) [16]. This fact agrees with previous studies conducted in other African countries [5,6,8,17]. However there are studies with a similar proportion of men/and women [18,19], and others where the ratio is as high as 3:1 [20,21]. In our study the ratio male/female in admitted leprosy patients was 1.8, however in a recent national demographic survey [22], carried on by the Ethiopian Ministry of Health, the amount of men in an aleatory sample that was representative of almost 18.000 households in the whole country was inferior to the amount of women. In Ethiopia and in Oromiya region, the ratio of men to women was 0.78 and 0.83, respectively, so, in our study, the rate male/female is not biased toward males.

Female patients admitted were younger than male patients. Arora et al. [20] in a study performed in cases diagnosed in a tertiary care centre in India found leprosy more prevalence in female than in male in the age group ranged 15 to 35 years, suggesting that hormonal imbalance related to pregnancy/puerperium, might play a role. This peak incidence observed in women in the fertile age group has also been reported in other studies [23].

Female patients live in the catchment area more often than male patients, though is not seen in the multivariate analysis. It might be explained by the reason that male patients have more incomes, and they are more likely to afford to travel further to get care. But, this question is not possible to answer with the type of design of this study.

The prevalence of MB leprosy cases admitted to hospital was lower in the female group than in the male one. However, the effect of WHO leprosy classification was not significant after adjustment for other variables including age. The prevalence of MB leprosy in male patients has been reported to be higher than in female ones in different series of new cases diagnosed in endemic countries such as Nigeria, Indonesia, Brazil, Nepal and Malawi [6,18-21].

Chronic skin ulcers are among the most serious complications of leprosy [24]. In our study, the main diagnosis during admission to hospital was neuropathic skin ulcer in lower extremities associated with infection or osteomyelitis. Neuropathic skin ulcers are one of the most common sequelae of leprosy and can result in large economic and social burden [25,26]. In our study, this diagnosis was less common in women than in men admitted to hospital in the univariate analysis. A descriptive cross-sectional study conducted in 245 leprosy patients with infected ulcers visiting three Ethiopian hospitals (ALERT, Kuyera and GRH) from August 2006 to May 2007 found an incidence of ulcers of 64.1% in men and 35.9% in women [24]. Similar results have been reported in other studies [27,28]. Britton and Lockwood [29] described this predominance, as true difference between men and women. This is not because of being underdiagnosed in women, but in some countries it was noticed by the delayed presentation of female patients, which results in high deformity.

Admissions for medical problems associated with leprosy, such as leprorreaction, or neuritis were similar in the female and male groups. In this regard, admissions for other diagnosis, apparently unrelated to leprosy, such as cardiovascular diseases (p.e. chronic heart failure or stroke) or gastroenteritis were more common in women than in men.

Leprosy per se is not fatal [30], death among active patients can be regarded as an unusual event, in our study approximately 3 percent died. Although the overall death rate in the sample was very low, we observed differences in fatality rates by gender. Fatal cases were more common in women than in men in multivariate analysis, finding that might be related to the comorbidity pattern leading to hospital admission.

Several limitations should be considered in this study. Due to the fact that it was retrospectively conducted with data collected from a registry book, some of our data are incomplete, including co-morbidities and neurological assessments. Moreover, the re-admission rate was recorded in less than two thirds of patients admitted.

## Conclusions

Despite these limitations, we understand that the characteristics of women admitted in our institution are different than in men, they were younger, had a different profile of admission and a higher mortality rate than male ones. However, with our results it is not able to get other conclusions about gender relation with the access to health care coverage, the delay in seeking for care and the isolation and rejection from the society.

## Competing interests

The authors declare that they have no competing interests.

## Authors’ contributions

IB did the literature review and wrote the introduction. FR and DL review the cases. JMR and MMM wrote the methods section and carried out the data analysis. JMR, MMM and FG wrote the Results and Conclusions sections. JMR, MMM, DL, IB, FG, read and approved the final manuscript.
